# Optimization of Prime Editing in Rice, Peanut, Chickpea, and Cowpea Protoplasts by Restoration of GFP Activity

**DOI:** 10.3390/ijms23179809

**Published:** 2022-08-29

**Authors:** Sudip Biswas, Aya Bridgeland, Samra Irum, Michael J. Thomson, Endang M. Septiningsih

**Affiliations:** 1 Department of Soil and Crop Sciences, Texas A&M University, College Station, TX 77843, USA; 2 Department of Biological Sciences, International Islamic University, Islamabad 44000, Pakistan

**Keywords:** prime editing, CRISPR-Cas9, dual pegRNA, mutant GFP, legume, editing efficiency

## Abstract

Precise editing of the plant genome has long been desired for functional genomic research and crop breeding. Prime editing is a newly developed precise editing technology based on CRISPR-Cas9, which uses an engineered reverse transcriptase (RT), a catalytically impaired Cas9 endonuclease (nCas9), and a prime editing guide RNA (pegRNA). In addition, prime editing has a wider range of editing types than base editing and can produce nearly all types of edits. Although prime editing was first established in human cells, it has recently been applied to plants. As a relatively new technique, optimization will be needed to increase the editing efficiency in different crops. In this study, we successfully edited a mutant GFP in rice, peanut, chickpea, and cowpea protoplasts. In rice, up to 16 times higher editing efficiency was achieved with a dual pegRNA than the single pegRNA containing vectors. Edited-mutant GFP protoplasts have also been obtained in peanut, chickpea, and cowpea after transformation with the dual pegRNA vectors, albeit with much lower editing efficiency than in rice, ranging from 0.2% to 0.5%. These initial results promise to expedite the application of prime editing in legume breeding programs to accelerate crop improvement.

## 1. Introduction

Precise gene editing is crucial for functional genomics studies and crop improvement [1,2]. Sequence deletion, insertion, and replacement have been performed by homology-directed repair (HDR) of double-stranded breaks (DSBs) through the presence of a donor DNA template [3]. Currently, using HDR in basic plant research and crop improvement is very limited because of its low efficiency and the difficulty of DNA template delivery [4]. However, this technique is still important in plant breeding for large sequence insertion/precise knock-in and complex DNA modification [5]. Base editing (BE) has been recognized as an alternative to HDR-mediated replacement and precise genome editing that greatly enhances crop breeding opportunities [6,7,8]. BE can perform up to 100-fold higher efficiency than HDR in obtaining the desired mutations [9]. Cytosine and adenine base editors (CBEs and ABEs) are the two widely used groups of base editors that can install C•G-to-T•A and A•T-to-G•C transitions, respectively [10]. For CBE, the Cas9 nickase (nCas9) or catalytically dead Cas protein (dCas9) is fused with a cytidine deaminase that converts the original C to T in the targeted DNA region [11]. In ABE, nCas9 or dCas9 is fused with adenosine deaminase, which permits A·T to G·C base substitutions in the target DNA sequence [12]. Both CBEs and ABEs have been well established in various crops, including rice, wheat, maize, tomato, and cotton [13,14,15,16,17].

Although base editors in plants are highly efficient, they are limited to only four types of base changes; however, manipulation of many agronomic traits may require the other eight nucleotide substitutions (A•T-to-C•G, C•G-to-A•T, T•A-to-A•T and G•C-to-C•G) and deletions or insertions [18]. A more recent technique, prime editing, can perform more efficient and precise genome editing through its ability to generate nearly any type of edit [19]. Thus, prime editing holds more promise for a wider scope of precise mutations for crop improvement once it is successfully optimized in each crop of interest. There are three prime editor systems (PPEs) available: PPE2, PPE3, and PPE3b. PPE2 consists of an nCas9 (H840A) fused to an engineered M-MLV reverse transcriptase (RT) and a pegRNA composed of a primer binding site (PBS) and an RT template. PPE3 adds nicking single- guide RNA (sgRNA) to cleave the non-edited strand, facilitating favorable DNA repair. In PPE3b, this nicking sgRNA targets the edited sequence, thereby preventing nicking of the non-edited strand until after editing occurs, resulting in fewer indels in mammalian cells [19]. In contrast to observations in mammalian cells, however, the editing efficiencies of the three PPEs were similar in plant cells [20].

Several studies using prime editing have recently been published across various plants, including rice, wheat, maize, potato, and tomato [3,20,21,22,23]. In wheat, the frequencies of single nucleotide substitutions, including A-to-T, C-to-G, G-to-C, T-to-G, and C-to-A, reached up to 1.4% [20]. Interestingly, using dual PEG and designing a perfect PBS, prime editing efficiency was increased up to 17% in rice [24]. Considering the usefulness of this technology, prime editing efficiency needs to be further improved in different crops. Along with this effort, plant protoplasts have been recognized as a useful platform for optimizing various gene editing techniques [25,26,27]. To our knowledge, prime editing in legumes has not been explored thus far. As it has been achieved in rice and several other crops, we hypothesize that it is possible to perform prime editing in legumes as well. This study aims to optimize prime editing in rice, peanuts, chickpeas, and cowpeas by transiently targeting mutant GFP in protoplasts. 

## 2. Results

### 2.1. Design of Mutant GFP and sgRNAs for PEG RNAs

**Figure 1:**
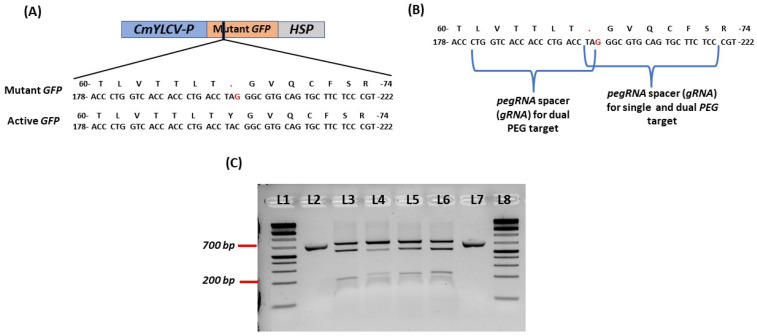
Design of mutant GFP and gRNAs. (**A**) map of mutant GFP; (**B**) position of pegRNA spacers/gRNAs for single pegRNA and dual pegRNAs; (**C**) in vitro RNP digestion of gRNAs using Cas9. L1 and L8: 1 kb+ ladders; L2 and L7: uncut amplicon from mutant GFP target region; L3: mutant GFP target region of sgRNA4 digested with Cas9 (expected bands of 531 bp and 190 bp); L4: mutant GFP region of sgRNA3 digested with Cas9 (expected bands of 518 bp and 203 bp); L5: mutant GFP region of sgRNA2 digested with Cas9 (expected bands of 522 bp and 199 bp); L6: mutant GFP region of sgRNA1 digested with Cas9 (expected bands of 521 bp and 200 bp).

To develop a test platform to measure prime editing efficiency, a stop codon (ATG) was inserted in the GFP coding region by changing C to G at the 202 position using overlapping PCR (Figure 1A,B). Four gRNAs were then designed using CRISPR-P2; their efficiency was tested by in vitro ribonucleoprotein (RNP) digestion of the PCR amplicons with Cas9 nuclease and synthetic gRNAs. All four of the gRNAs cut the target GFP sequence efficiently (Figure 1C). Two of the four gRNAs were used for pegRNA design because of the close proximity of the mutation site.

### 2.2. Development of Mutant GFP Vector and Testing in Protoplasts

**Figure 2:**
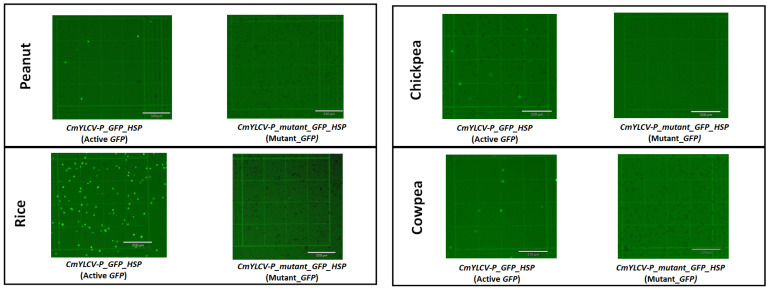
Testing of active GFP and mutant GFP cloning vectors in peanut, rice, chickpea, and cowpea protoplasts via PEG transformation. Micrographs of protoplasts expressing active GFP and mutant GFP under the fluorescent field are shown. The scale bar length for all the pictures was 320 µm. Micrographs under the bright field are shown in Supplementary Figure S1.

An expression vector containing the mutant GFP sequence driven by the CmYLCV promoter was developed. After introducing a stop codon in the GFP coding sequence, the mutant GFP was inserted into the module A vector by removing the active GFP through restriction digestion cloning. The mutant GFP vector was then tested in rice, peanut, chickpea, and cowpea protoplasts, along with the active GFP expression vector as a control. At 48 h after transformation, no GFP expression was observed in the protoplasts with mutant GFP. In contrast, a high level of GFP expression was seen in the rice, peanut, chickpea, and cowpea protoplasts with the active GFP vector (Figure 2). The results showed that the activity of the GFP was successfully terminated by the insertion of the stop codon (TAG).

### 2.3. Testing of Prime Editing Vectors in Rice, Peanuts, Chickpeas, and Cowpeas

**Figure 3:**
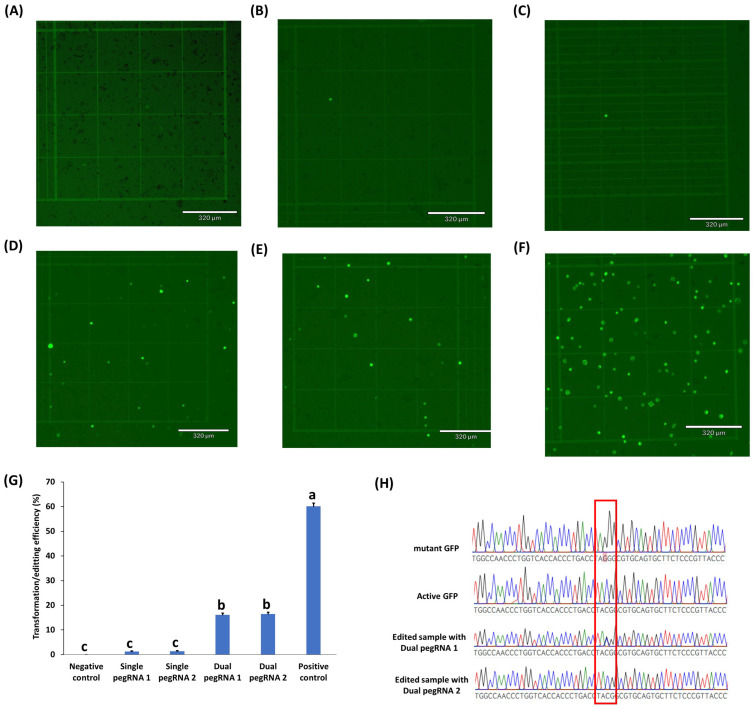
Prime editing in rice protoplasts transformed using single or dual pegRNAs containing vectors. (**A**) negative control (no GFP plasmid/prime editing vectors); (**B**) protoplasts with single pegRNA1 containing vector; (**C**) protoplasts with single pegRNA2 containing vector; (**D**) protoplasts with dual pegRNA1 containing vector; (**E**) Protoplasts with dual pegRNA2 containing vector; (**F**) positive control (protoplasts with CmYLCV_GFP vector); (**G**) the transformation efficiency (TE) of protoplasts transformed with different prime editing vectors. TE was evaluated after incubation in 40% PEG solution with 20 µg plasmid DNA of each prime editing vector. Values represent means ± SE (n = 6). The different letters indicate significant differences at *p* < 0.05; (**H**) Sanger sequencing results of active GFP, mutant GFP, and samples transformed by the dual pegRNAs containing vectors. Scale bar length for all pictures was 320 µm. Micrographs under the bright field are shown in Supplementary Figure S3.

Four different prime editing vectors were used to test the efficiency of prime editing to restore the functional activity of the GFP mutant in rice, peanut, chickpea, and cowpea protoplasts: vectors with single pegRNA1, single pegRNA2, dual pegRNA1, and dual pegRNA2 (Supplementary Figure S2). Their GFP expression was evaluated at 24h post transformation (Figure 3). Both of the single pegRNAs containing vectors showed low GFP expression in rice protoplasts (Figure 3B,C,G). In contrast, both of the dual pegRNAs containing vectors gave significantly higher expression than the single pegRNAs vectors (Figure 3D,E,G). This result demonstrated that dual pegRNAs vectors had 16 times higher prime editing efficiency than the single pegRNAs vectors in rice. There was no GFP expression in the negative control (Figure 3A). On the other hand, higher GFP expression/transformation efficiency (60%) was observed in rice protoplasts transformed with the CmYLCV GFP expression vector (Figure 3F). Succesful edits of mutant GFP (G to C) using either of the dual pegRNAs containing vectors in rice protoplasts were further confirmed through Sanger sequencing (Figure 3H). 

**Figure 4:**
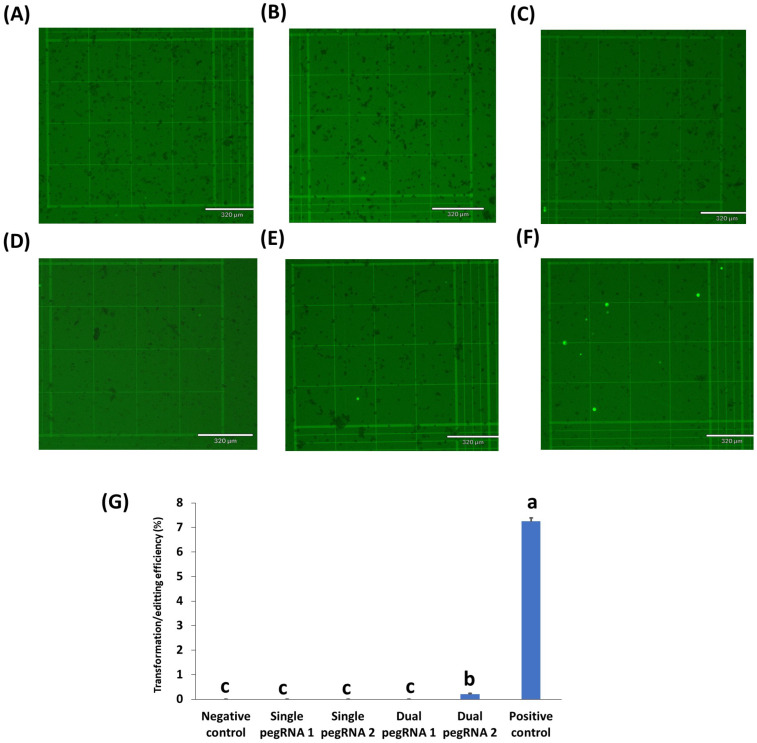
Prime editing in peanut protoplasts transformed using single or dual pegRNAs containing vectors. (**A**) negative control (no GFP plasmid/prime editing vectors); (**B**) protoplasts with single pegRNA1 containing vector; (**C**) protoplasts with single pegRNA2 containing vector; (**D**) protoplasts with dual pegRNA1 containing vector; (**E**) protoplasts with dual pegRNA2 containing vector. (**F**) positive control (protoplasts with CmYLCV_GFP vector). (**G**) the transformation efficiency (TE) of protoplasts transformed with different prime editing vectors. TE was evaluated after incubation in 50% PEG solution with 300 µg plasmid DNA of each prime editing vector. Values represent means ± SE (n = 6). The different letters indicate significant differences at *p* < 0.05. Scale bar length for all pictures was 320 µm. Micrographs under the bright field are shown in Supplementary Figure S4.

GFP expression was also evaluated at 24 h post transformation in peanut protoplasts (Figure 4). Unfortunately, there was no GFP expression in the peanut protoplasts with the two single pegRNAs and the dual pegRNA1 containing prime editing vectors (Figure 4B–D). Nonetheless, the dual pegRNA2 vector where all the genes (nCAS9-M_MLV, dual pegRNA, and mutant GFP) were expressed by the CmYLCV promoter showed some lower GFP expression (Figure 4E,G). As expected, there was no GFP expression in the negative control (Figure 4A), whereas there was a reasonable level of GFP expression (7%) in the peanut protoplasts with the positive control (CmYLCV_GFP) (Figure 4F).

**Figure 5:**
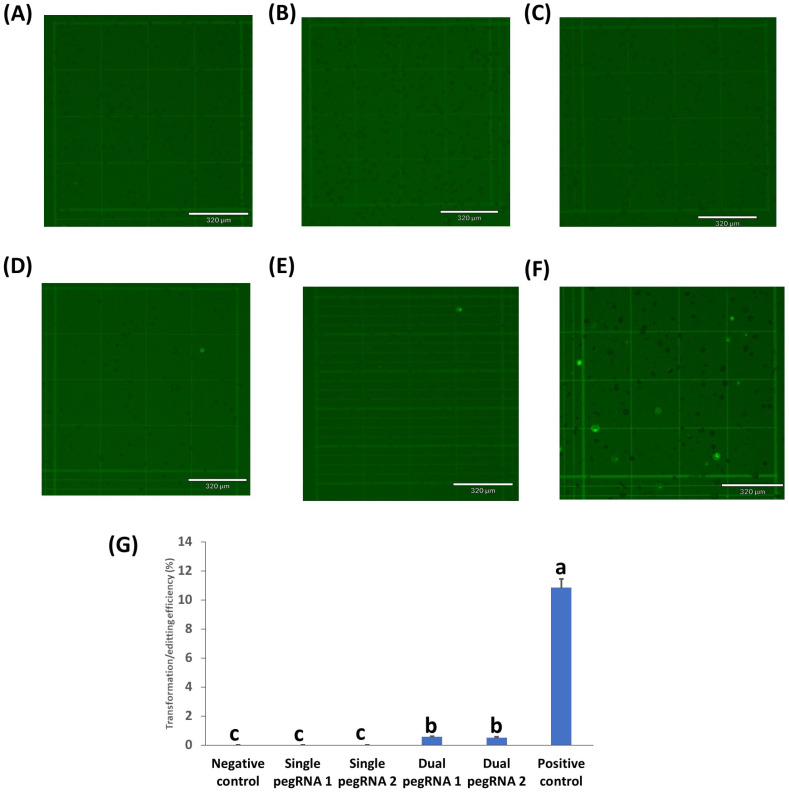
Prime editing in chickpea protoplasts transformed using single or dual pegRNAs containing vectors. (**A**) negative control (no GFP plasmid/prime editing vectors); (**B**) protoplasts with single pegRNA1 containing vector; (**C**) protoplasts with single pegRNA2 containing vector; (**D**) protoplasts with dual pegRNA1 containing vector; (**E**) protoplasts with dual pegRNA2 containing vector; (**F**) positive control (protoplasts with CmYLCV_GFP vector); (**G**) transformation efficiency (TE) of protoplasts transformed with different prime editing vectors. TE was evaluated after incubation in 50% PEG solution with 60 µg plasmid DNA of each prime editing vector. Values represent means ± SE (n = 6). The different letters indicate significant differences at *p* < 0.05. Scale bar length for all pictures was 320 µm. Micrographs under the bright field are shown in Supplementary Figure S5.

**Figure 6:**
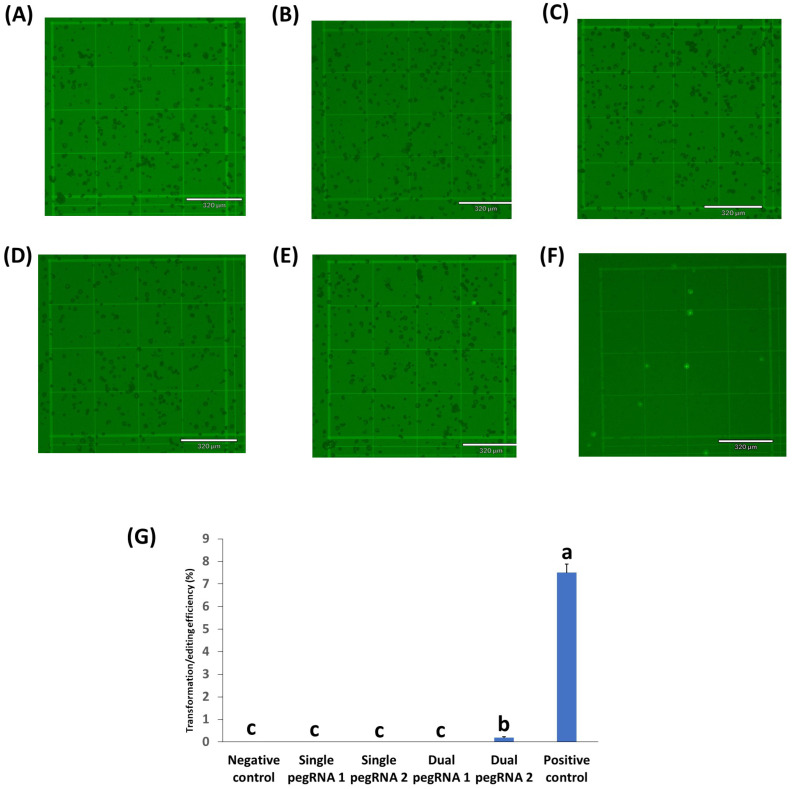
Prime editing in cowpea protoplasts transformed using single or dual pegRNAs containing vectors. (**A**) negative control (no GFP plasmid/prime editing vectors); (**B**) protoplasts with single pegRNA1 containing vector; (**C**) protoplasts with single pegRNA2 containing vector. (**D**) protoplasts with dual pegRNA1 containing vector; (**E**) protoplasts with dual pegRNA2 containing vector; (**F**) positive control (protoplasts with CmYLCV_GFP vector); (**G**) transformation efficiency (TE) of protoplasts transformed with different prime editing vectors. TE was calculated after 24 h and incubation in 50% PEG solution for 10 min with 100 µg plasmid DNA of each prime editing vector. Values represent means ± SE (n = 6). The different letters indicate significant differences at *p* < 0.05. Scale bar length for all pictures was 320 µm. Micrographs under the bright field are shown in Supplementary Figure S6.

GFP expression was also tested in chickpeas and cowpeas (Figure 5 and Figure 6). In the case of chickpeas, both of the dual pegRNAs containing vectors showed lower GFP expression in the protoplasts (Figure 5D,E). However, only the dual pegRNA2 containing vector gave GFP expression in the cowpeas, similar to peanut protoplasts (Figure 6E). There was no expression observed in single pegRNAs containing vectors in both chickpeas and cowpeas. As expected, no expression was detected on the negative control (Figure 5A and Figure 6A), but a reasonable amount of GFP expression was observed after transformation with positive control in chickpeas and cowpeas (Figure 5F and Figure 6F).

## 3. Discussion

The CRISPR-Cas9 system has revolutionized the field of agriculture in the last decade; nevertheless, precise genome editing remains a major challenge. In plants, homology-directed repair is still limited due to low efficiency and challenges in delivering the template DNA to make precise edits [28]. The first set of base editors, cytosine and adenine base editors (CBEs and ABEs), also have several drawbacks, including lower efficiency, the possibility of off-target mutation effects, and the limited capability in editing only four types of base changes [29]. Prime editing, however, has a more versatile capability for broader applications in crop improvement by making more precise edits through insertions, deletions, and substitutions with all possible combinations of bases [20]. 

Testing of the CRISPR-Cas system can be performed relatively quickly in protoplasts due to the convenience of protoplast isolation and transfection in different plant species [30]. In addition to plasmid transformation, recent studies have shown success in using ribonucleoprotein (RNP) delivery into protoplasts using PEG-mediated delivery, lipofection, or electroporation for CRISPR-Cas editing across diverse crops, including maize, tomatoes, cabbage, and chickpeas, among others [31,32,33,34,35]. Moreover, transient expression in protoplasts can be used for validation of Cas codon optimization or modification, sgRNA validation, selecting the most efficient promoter, and analysis of different vector designs [26,36]. Drawbacks of using protoplasts for gene editing include the difficulty of regenerating whole plants from the protoplasts, problems with somaclonal variation, and challenges in selecting edited cells without the use of selectable markers [35,36]. Protoplast protocols also need to be optimized for each species. For instance, varying PEG concentrations and incubation times need to be tested to obtain high transformation rates without killing the protoplasts. After optimization, we obtained a 39–52% survival rate after PEG mediated transformation in the four target species (Supplementary Figures S7 and S8), while transformation efficiencies ranged from 7–10% in the legumes and 60% in the rice in our study. Once optimized protocols are in place, protoplasts offer an efficient system to rapidly test gene editing components in vivo. Therefore, protoplasts present the ideal platform for the determination of prime editing vector efficiency in a relatively short period of time.

Although low editing efficiency limited the first prime editing studies, more recently, up to 17% editing efficiency was obtained in rice protoplast transformation by using two prime editing guide (peg) RNAs (dual PEG) in trans direction for the same target [24]. In this study, we successfully developed single and dual PEG vectors for prime editing via Golden Gate assembly and demonstrated their efficacy in rice and several crops in the legume family. In our study on rice, where up to 60% transformation efficiency was obtained with the positive control (35S_GFP plasmid), higher editing efficiency (16%) was achieved similarly to previously published data [24], as confirmed with Sanger sequencing. However, a much lower percentage of edits in peanuts, chickpeas, and cowpeas was observed (0.2–0.5%, CmYLCV_GFP), which might be due in part to the lower overall protoplast transformation efficiency in these legume protoplasts, as seen with the positive control, which is about 7–10%. 

In this study, three different promoters were used: CAMV 35S, CmYLCV, and OsU6. These promoters worked well in the rice protoplasts, but only the CmYLCV promoter provided up to 7–10% transformation efficiency in the peanuts, chickpeas, and cowpeas. This indicated that the type of promoter significantly contributes to the success of prime editing. Other crucial parameters are sgRNA position for nCas9, and reverse transcriptase (RT) and primer binding site (PBS) length [24]. 

Prime editing has been demonstrated in several crop species, but to our knowledge has not yet been investigated in legume species. The initial prime editing reports focused on monocot species: after the original prime editing protocol was published, several studies demonstrated prime editing in rice [3,20,21,37,38,39], wheat [20], and maize [40]. Prime editing was also shown effective in a dicot in a study using tomatoes [23]. Prime editing efficiencies have generally been low, with initial rice studies showing efficiencies of 0.26% to 2% in calli [38], 2.2% to 9.4% in calli [37], 2% to 8.2% in protoplasts [20], and 9% in transgenic plants [39]. Moreover, wheat protoplasts only had up to 1.2% efficiency, while transgenic maize plants had efficiencies of 6.5% and 53.2% at two ALS gene targets [40]. For the single dicot study, luciferase assays in tomato leaves showed efficiencies of 0.26% to 2.6%, regenerated shoots had edits in 0.025% to 1.66% of NGS reads, and transgenic tomato plants ranged from 3.4% to 6.7% with the targeted edits but were chimeric [23]. Several recent reports have engineered optimized prime editing systems, with editing efficiency increased from 2.1% to 11.3% [41] and 2.9% to 17.4% [24] in rice protoplasts, maize protoplasts up to 6.2% [42], and transgenic rice plants up to 24.3% prime editing efficiency [42]. Reported prime editing efficiencies in transgenic plants have often been higher than in protoplasts, which may be due to a greater chance for edits in transgenic plants as the cells pass through multiple cycles of division. Thus, while our study showed a low range of prime editing efficiency in legumes, ranging from 0.2% to 0.5% of protoplast cells showing the targeted edits, a higher editing efficiency is expected once transgenic plants are developed. Moreover, further optimization of the prime editing system should improve editing efficiency in legumes. 

## 4. Materials and Methods

### 4.1. Plant Materials

The temperate japonica rice cultivar Nipponbare, the peanut cultivar Schubert [29], an elite cowpea breeding line IT97K-499-35 [43], and the chickpea cultivar Kocbasi (Kabuli type) were used. All the plant seedlings were grown in a greenhouse with a temperature of 32/26 °C (day/night) and a 16/8 h light-dark cycle.

### 4.2. Prime Editing Vector Construction

CmYLCV_GFP_HSP and 35S_GFP_NOS vectors were used for active GFP expression in protoplasts. Three intermediate module plasmids A, B, and C, and one backbone vector, pTRANS_100, were employed to develop the prime editing vectors [44] (Supplementary Figure S2). A mutant GFP vector was generated by changing C to G at 202 positions to produce a new stop codon (TAG) in the coding sequence. This was performed using overlapping PCR and then cloning them into a CmYLCV_GFP_HSP vector by removing the active GFP sequence through restriction digestion cloning with a T4 DNA ligase (NEB, Ipswich, MA, USA). CmYLCV_mutant_GFP_HSP was prepared as Module A (Supplementary Figure S2A). For making pegRNAs, four gRNAs were designed in the mutant GFP position. The efficiency of the gRNAs was checked using the in vitro ribonucleoprotein (RNP) digestion of DNA with Cas9 Nuclease (NEB, Ipswich, MA, USA) provided by the manufacturer with a few modifications. In this case, a 27 μL reaction mixture containing 30 nM of synthesized sgRNA (Synthego, Redwood City, CA, USA), 30 nM of Cas9 nuclease, and 3 μL of 10× NEB buffer 3.1 was pre-incubated for 10 min at 25 °C. Afterward, a 100 ng purified PCR product was added to make a total reaction volume 30 μL, incubated at 37 °C for 1h. After adding 1 μL of Proteinase K, the reaction mixture was kept for 10 min at 56 °C, and fragment analysis was performed using gel electrophoresis.

For the mutant GFP target, two single pegRNAs were designed using pegFinder [45], and one dual pegRNA was designed using PlantPegDesigner [24]. An endogenous tRNA processing system was used for dual pegRNA expression [46]. All the pegRNAs were synthesized and cloned into pMOD_2515b/pMOD_B2303, where the pegRNAs were driven by the OsU6/CmYLCV promoter (Supplementary Figure S2B). The nCAS9 and M-MLV RT were amplified from the nCas9-PPE plasmid (Addgene #140445) and cloned into the 35S_GFP_NOS vector by removing the GFP. For making the CmYLCV_ nCAS9 + M-MLV _NOS, the CmYLCV was placed by removing the 35S promoter from 35s_ nCAS9 + M-MLV _NOS plasmid (Supplementary Figure S2C). The CmYLCV_mutant_GFP_HSP (module A), pMOD_2515b/pMOD_B2303_pegRNA (Module B), and 35S/CmYLCV_nCAS9+M-MLV_NOS (Module C) were cloned into a non-binary pTRANS_100 through Golden Gate assembly cloning [31] (Supplementary Figure S2D). 

### 4.3. Protoplast Isolation and Transfection

Rice protoplasts were isolated from the stems of 10–12-day-old rice seedlings according to established protocols [47,48] with some modifications. Briefly, the stems and sheaths of ~30 rice seedlings per trial (total 100–120 seedlings) were cut into latitudinal strips. The strips were transferred into a 150-mL conical flask containing 50 mL of filter-sterilized enzyme solution (1.5% (*w*/*v*) Cellulase RS, 0.1% (*w*/*v*) Macerozyme R-10, 0.4 M Mannitol, 20 mM KCl and 20 mM MES (pH 5.7)), and the flask was wrapped with aluminum foil. The strips with cell wall–digesting enzymes were vacuum-infiltrated by applying a vacuum (~380–508 mmHg) for 30 min in the dark. Next, the strips were incubated in the dark for 5 h with gentle shaking (50 rpm) at room temperature. After enzymatic digestion, 50 mL of W5 solution (154 mM NaCl, 125 mM CaCl_2_, 5 mM KCl, and 2 mM MES (pH 5.7)) was added to the conical flask and shaken gently by hand for 10 s to release the protoplasts. The protoplasts were collected into three or four 50-mL round-bottomed centrifuge tubes after filtering the mixture through a 40-µm nylon mesh and washing the strips on the surface of the nylon mesh 3–5 times with W5 solution. The solution containing protoplasts was centrifuged at 250 *g* for 3 min at room temperature (RT) in a swinging bucket rotor, and the supernatant was removed by pipetting. The protoplasts were resuspended in 10 mL of W5 solution, collected into a 50-mL round-bottomed tube, and centrifuged at 250× *g* for 3 min at room temperature. The supernatant was then removed by pipetting, and the protoplasts were resuspended in 4 mL of MMG solution (0.4 M Mannitol, 15 mM MgCl_2_ and 4 mM MES (pH 5.7)). The concentration of the protoplasts was determined under a microscope (×100) with a hemocytometer. Rice protoplast transfection with the prime editing vectors was performed using PEG (polyethylene glycol) according to Shan et al. [48]. Peanut protoplast isolation and transformation were performed according to our established protocol [26]. For the chickpea and the cowpea, the procedures followed previously published protocols with some modifications [49,50]. An approximately similar quantity of protoplasts (2 × 10^6^ total cells) was used from each species for each experiment (Supplementary Figure S7). For the peanut, the condition was 50% PEG, 5 min PEG incubation time at 13 °C, and 250 µg plasmid DNA of each vector. For the rice, the condition was 40% PEG, 20 min PEG incubation time at room temperature (25 °C), and 20 µg plasmid DNA of each vector. For the chickpea, the condition was 50% PEG, 10 min PEG incubation time at 4 °C, and 60 µg plasmid DNA of each vector. For the cowpea, the condition was 50% PEG, 10 min PEG incubation time at 13 °C, and 100 µg plasmid DNA of each vector.

### 4.4. Microscopy Analysis

The total, viable, and GFP-expressed protoplasts were counted with an ECHO Revolve 4 revolving microscope under normal light and fluorescent light (Bico Company, San Diego, CA, USA). The total number of protoplasts was counted under the microscope (×100) using a hemocytometer (XB. K.25, QiuJing, Shanghai, China). The protoplast density was calculated as follows: protoplast number (g − 1) = the average count of protoplast per square × 104. Fluorescein diacetate (FDA) and propidium bromide staining (Sigma-Aldrich, St. Louis, MO, USA) were used to determine the protoplast viability according to the manufacturer’s protocol. We used 320–340 nm wavelength to capture the images of the protoplasts. The transformation efficiency of each prime editing vector was calculated after 24 h from transformation. 

### 4.5. Mutant Analysis

After 4–5 days post transfection under dark conditions, the protoplasts were collected by centrifugation at 13,000 rpm. RNA was extracted following the protocol of the Zymo plant RNA isolation kit (Zymo, Irvine, CA, USA). Next, cDNA was synthesized according to the manufacturer’s protocol (RevertAid First Strand cDNA Synthesis Kit, ThemoFiser Scientific, Waltham, MA, USA). The targeted edited region of GFP was amplified with the Phusion Taq polymerase by primer sets (Forward_GFP: 5′-GTCCCAATTCTTGTTGAATTAGATG-3′ and reverse GFP: 5′-ACAGGTAATGGTTGTCTGGTAAAAG-3′) with an initial denaturation step of 98 °C for 30 s, followed by 30 cycles of 98 °C for 30 s, 58 °C for 30 s, and 72 °C for 30 s, and a final extension of 72 °C for 7 min. PCR products of GFP were purified by gel extraction and cloned into a TOPO vector (ThemoFiser Scientific, Waltham, MA, USA). Positive clones were sequenced through Sanger sequencing (Eurofins, Lancaster, PA, USA). 

## 5. Conclusions

This study has prepared the foundation for more precise edits and a possible path for allele replacement to accelerate crop improvement in legumes. Legumes have great potential to address many of the challenges of crop production, as they contribute to soil health through nitrogen fixation, provide essential proteins and fats for human nutrition, and can be bred to be stress-tolerant to provide a climate-resilient crop for the future. Genome editing has been successfully performed in at least four legume crops, namely soybeans, peanuts, cowpeas, and chickpeas, but is largely limited to gene knockouts [51,52]. Although there are no current publications demonstrating prime editing in legume crops, the current study shows promise that prime editing can be achievable in peanuts, cowpeas, and chickpeas. With further optimization, these results promise to enable more precise editing modifications in key traits for legume crops to meet the challenges of the future.

## Data Availability

Not applicable.

## Supplementary Materials

The following supporting information can be downloaded at: https://www.mdpi.com/article/10.3390/ijms23179809/s1.
