# Supplementary material for: Optimization of Prime Editing in Rice, Peanut, Chickpea, and Cowpea Protoplasts by Restoration of GFP Activity
Source: Int J Mol Sci. 2022 Aug 29;23(17):9809. doi: 10.3390/ijms23179809 (PMC9456013; doi:10.3390/ijms23179809)
Supplement: Supplementary file 1 [file ijms-23-09809-s001.zip › ijms-1821149-supplementary.pdf]

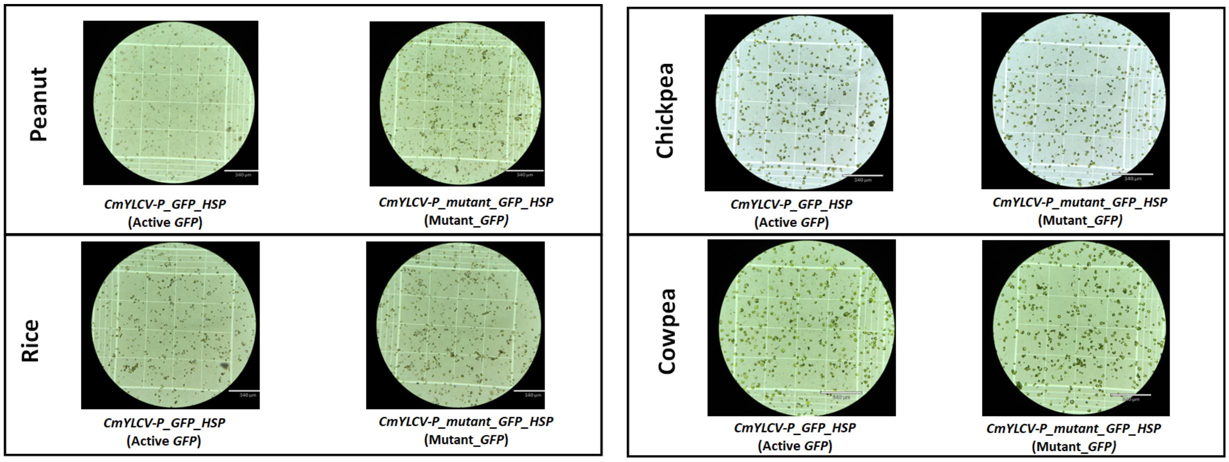

Supplementary Figure S1: Bright field micrographs of protoplasts from peanut, rice, chickpea and cowpea after PEG mediated transformation with active GFP and mutant GFP cloning vectors.

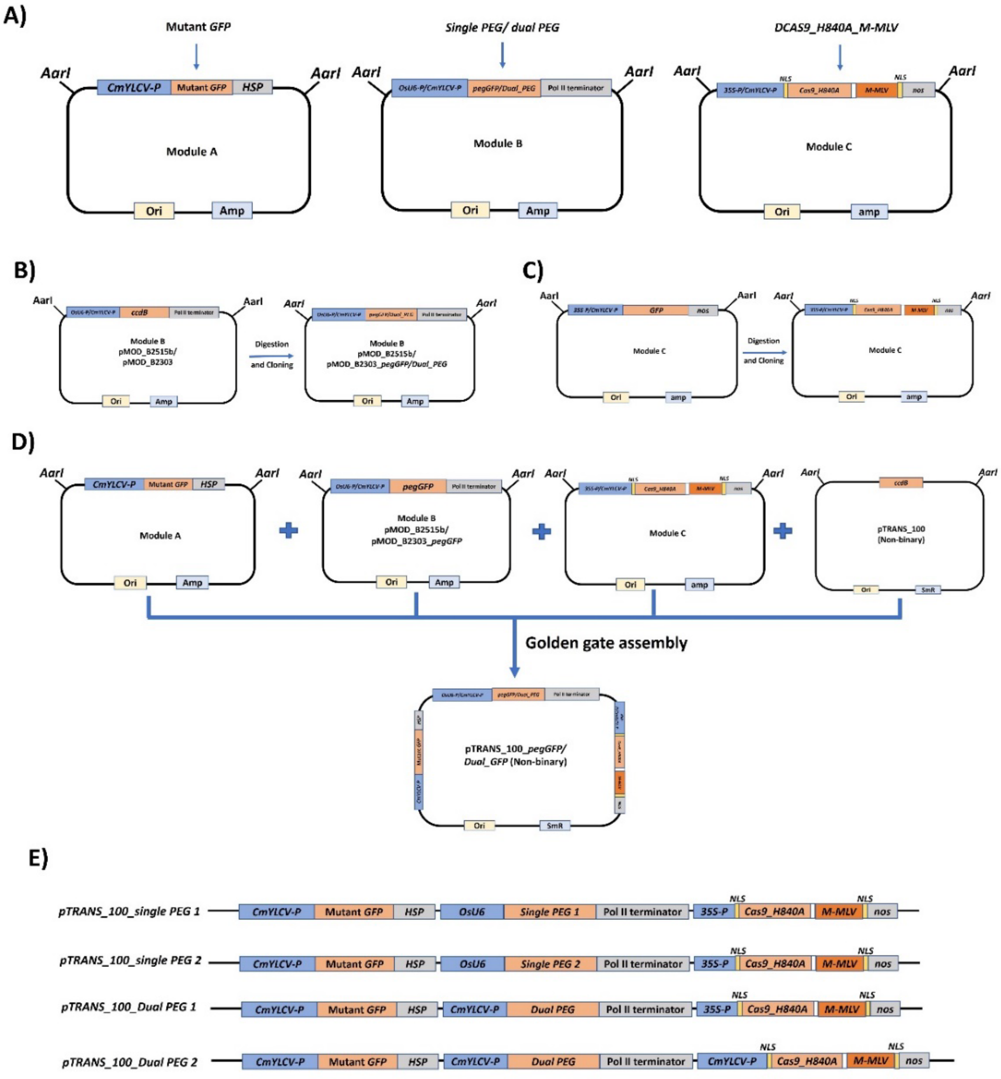

Supplementary Figure S2: Schematic diagram of cloning strategies of the prime editing vector in this study. A) Vector Modules used for the prime editing vector, CmYLCV-P_mutant_GFP_HSP was used as module A, OsU6-P/CmYLCV-P_single PEG/dual PEG_Pol II terminator was used as module B and 35S-P/CmYLCV-P_nCAS9_M_MLV_NOS was used as module C; B) Cloning of OsU6-P/CmYLCV-P_single PEG/dual PEG_Pol II terminator vector; C) Cloning of 35S-P/CmYLCV-P_nCAS9_M_MLV_NOS; D) Making of prime editing vectors using golden gate assembly; E) Schematic representation of different prime editing vectors used in this study.

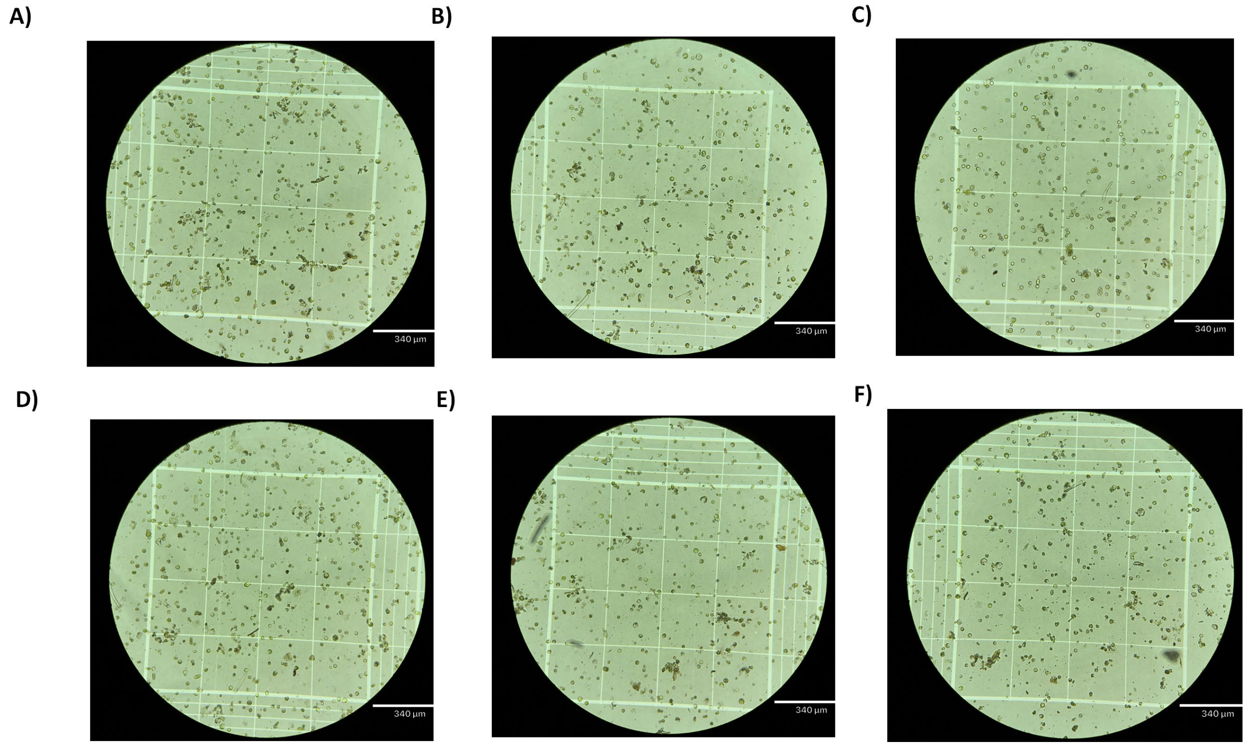

Supplementary Figure S3: Micrographs of prime editing in rice protoplasts transformed using single or dual pegRNAs containing vectors under the bright field. A) Negative control (no GFP plasmid/prime editing vectors); B) Protoplasts with single pegRNA1 containing vector; C) Protoplasts with single pegRNA2 containing vector; D) Protoplasts with dual pegRNA1 containing vector; E) Protoplasts with dual pegRNA2 containing vector; F) Positive control (protoplasts with CmYLCV_GFP vector).

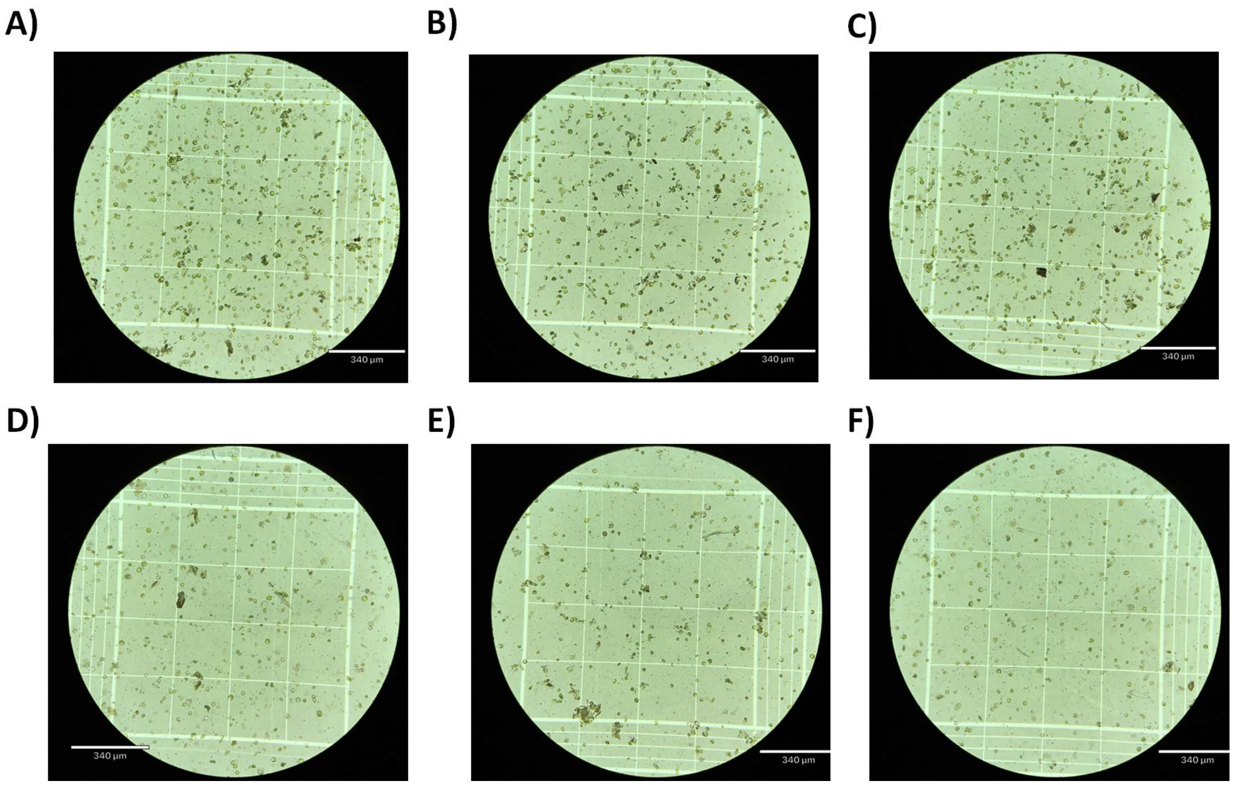

Supplementary Figure S4: Micrographs of prime editing in peanut protoplasts transformed using single or dual pegRNAs containing vectors under the bright field. A) Negative control (no GFP plasmid/prime editing vectors); B) Protoplasts with single pegRNA1 containing vector; C) Protoplasts with single pegRNA2 containing vector; D) Protoplasts with dual pegRNA1 containing vector; E) Protoplasts with dual pegRNA2 containing vector; F) Positive control (protoplasts with CmYLCV_GFP vector).

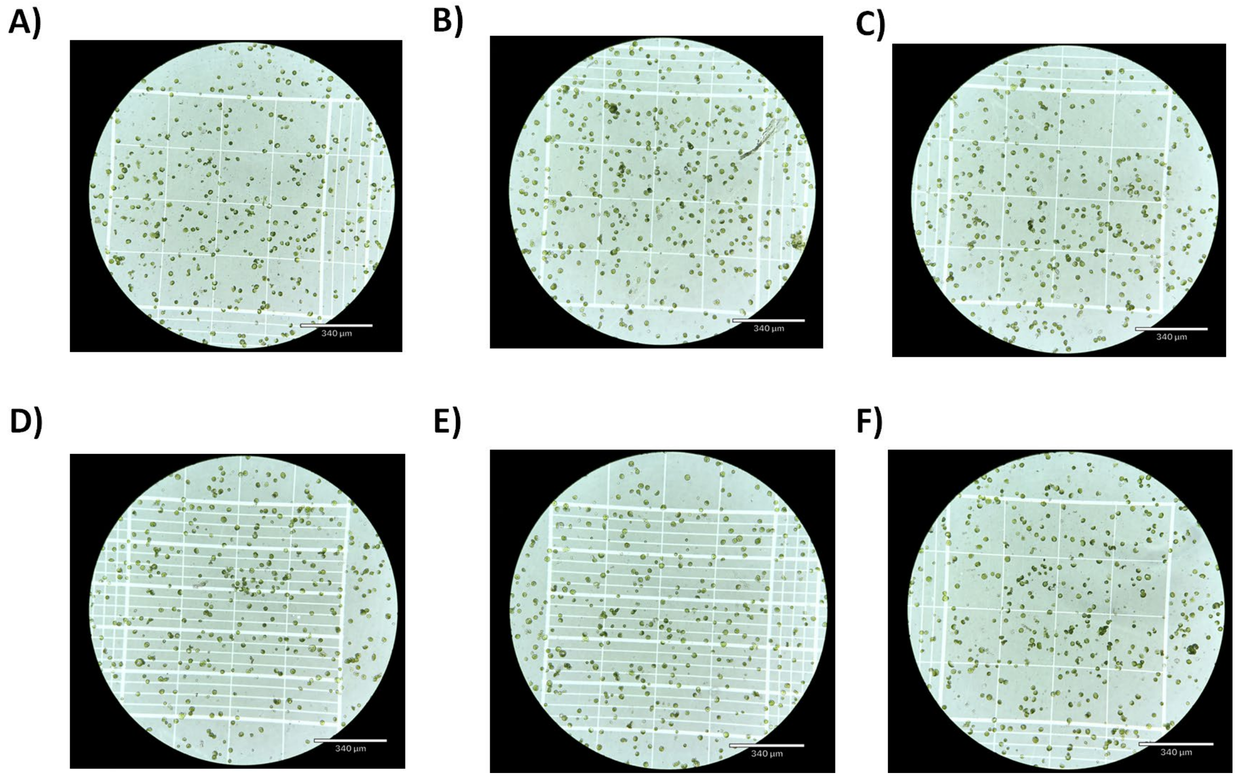

Supplementary Figure S5: Micrographs of prime editing in chikpea protoplasts transformed using single or dual pegRNAs containing vectors under the bright field. A) Negative control (no GFP plasmid/prime editing vectors); B) Protoplasts with single pegRNA1 containing vector; C) Protoplasts with single pegRNA2 containing vector; D) Protoplasts with dual pegRNA1 containing vector; E) Protoplasts with dual pegRNA2 containing vector; F) Positive control (protoplasts with CmYLCV_GFP vector).

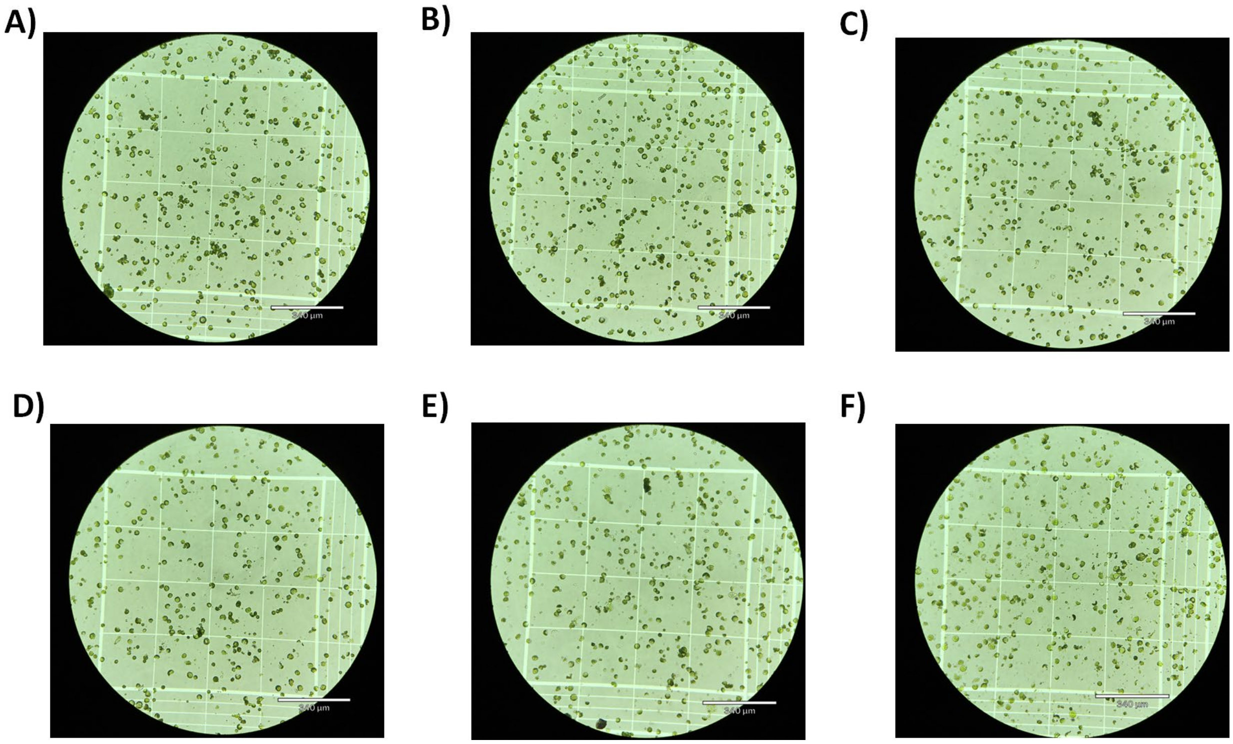

Supplementary Figure S6: Micrographs of prime editing in cowpea protoplasts transformed using single or dual pegRNAs containing vectors under the bright field. A) Negative control (no GFP plasmid/prime editing vectors); B) Protoplasts with single pegRNA1 containing vector; C) Protoplasts with single pegRNA2 containing vector; D) Protoplasts with dual pegRNA1 containing vector; E) Protoplasts with dual pegRNA2 containing vector; F) Positive control (protoplasts with CmYLCV_GFP vector).

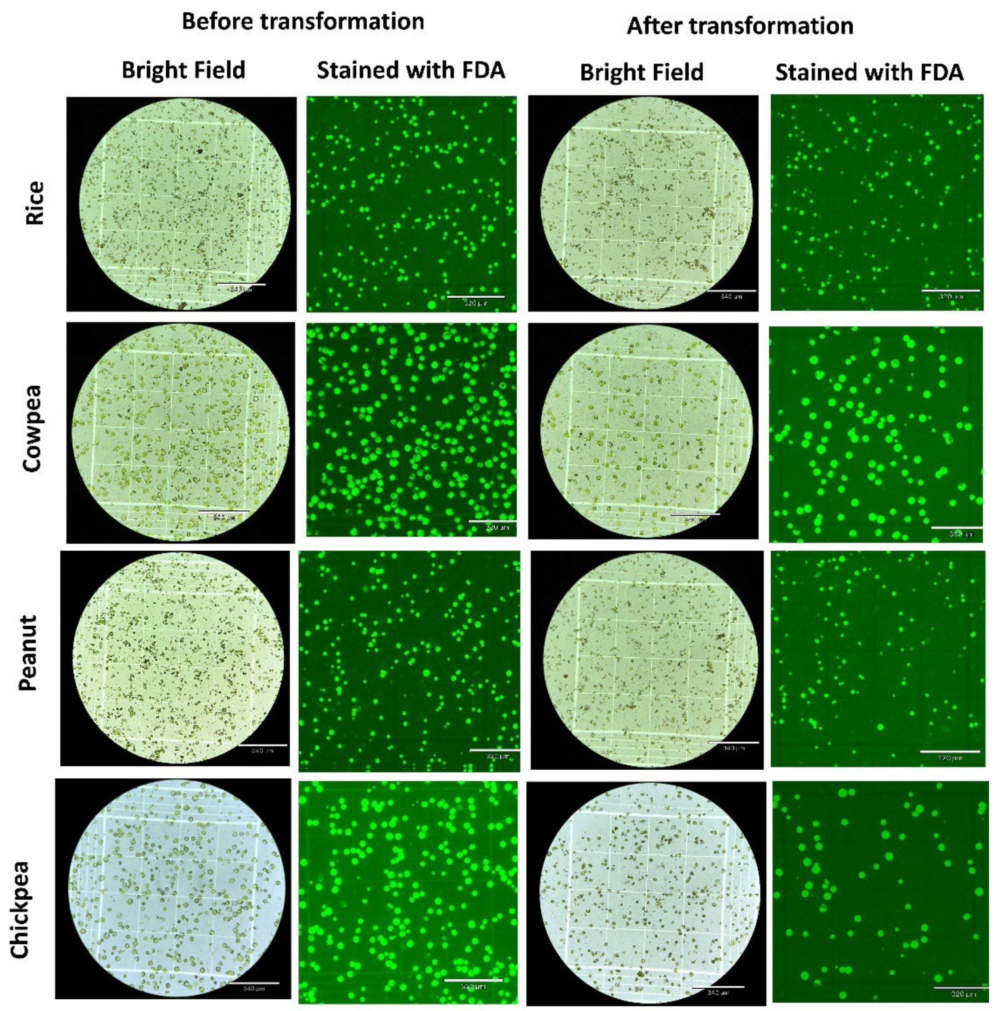

Supplementary Figure S7: Photograph of protoplasts from rice, cowpea, peanut and chickpea before and after transformation. Similar amount of protoplast (2×10^6 total cells) were used for every experiment.

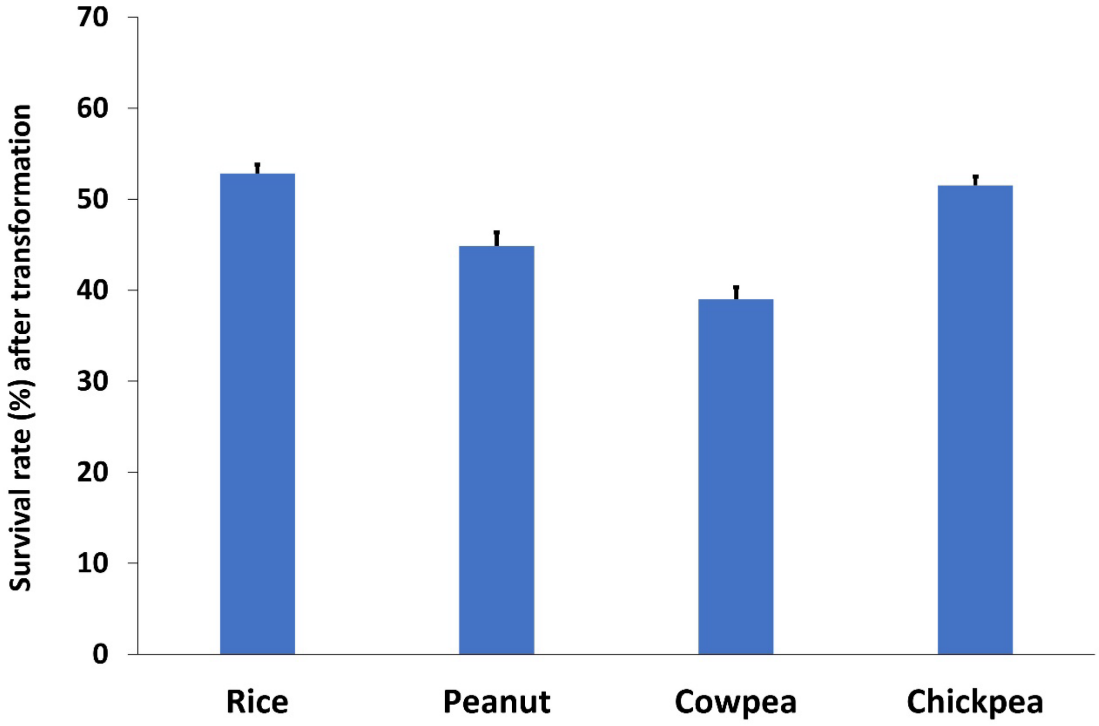

Supplementary Figure S8: Survival rates of protoplasts from rice, cowpea, peanut and chickpea after PEG mediated transformation.
